# Synthesis and characterization of an innovative sodium alginate/polyvinyl alcohol bioartificial hydrogel for forward-osmosis desalination

**DOI:** 10.1038/s41598-024-58533-6

**Published:** 2024-04-08

**Authors:** Menatalla Ashraf Saad, Eman Radi Sadik, Basma Mohamed Eldakiky, Hanan Moustafa, Eman Fadl, Zhen He, Elsayed Zakaria Elashtoukhy, Randa Eslah Khalifa, Taghreed Mohamed Mohamed Zewail

**Affiliations:** 1 Chemical Engineering Department, Faculty of Engineering, Alexandria University, Alexandria, 21544 Egypt; 2 Polymer Materials Department, Advanced Technologies and New Materials Research Institute (ATNMRI), City of Scientific Research and Technological Applications (SRTA City), P.O. Box: 21934, New Borg El-Arab City, Alexandria Egypt; 3 Biotechnology Department, Institute of Graduate Studies and Research, Alexandria University, Alexandria, 21526 Egypt; 4 Materials Science Department, Institute of Graduate Studies and Research, Alexandria University, Alexandria, 21526 Egypt; 5 Chemical Engineering Department, Borg Al Arab Higher Institute of Engineering and Technology, Alexandria, 21933 Egypt; 6 Department of Energy, Environmental and Chemical Engineering, Washington University in St. Louis, St. Louis, MO 63130 USA

**Keywords:** Bioartificial hydrogel, Draw agent, Forward osmosis, Desalination, Chemical engineering, Environmental chemistry, Energy, Materials chemistry, Organic chemistry, Chemical synthesis

## Abstract

Recently, hydrogels have been widely applied as draw agents in forward osmosis (FO) desalination. This work aims to synthesize bioartificial hydrogel from a blend of sodium alginate (SA) and polyvinyl alcohol (PVA) using epichlorohydrin (ECH) as a crosslinker. Then this prepared hydrogel was applied as a draw agent with cellulose triacetate membrane in a batch (FO) cell. The effects of the PVA content in the polymer blend and the crosslinker dose on the hydrogel’s swelling capacity were investigated to optimize the hydrogel’s composition. Furthermore, the water flux and the reverse solute flux of the optimum SA/PVA hydrogel were evaluated in a batch (FO) unit under the effect of the hydrogel’s particle size, feed solution (FS) temperature, FS concentration, and membrane orientation. Fourier transform infrared spectroscopy (FTIR), scanning electron microscopy (SEM), X-ray diffraction (XRD) and compression strength tests were used to characterize the prepared hydrogel. Results revealed that the equilibrium swelling ratio (%) of 5228 was achieved with a hydrogel that had 25% PVA and a crosslinking ratio of 0.8. FO experiments revealed that the maximum water flux of 0.845 LMH achieved, when distilled water was used as FS, average hydrogel’s particle size was 60 µm, and the FS temperature was 40 °C.

## Introduction

Water is the most valuable resource on earth for survival^1^. In view of the exponential growth of the human population, the world strongly suffers from a lack of water and energy resources^2^. In addition, the contamination of the current fresh water resources (such as freshwater lakes and groundwater) by household and industrial discharges exacerbates the scarcity of fresh water^3^. One of the most serious global water challenges is that nearly 2.8 billion people will be living in water-scarce or water-stressed regions of the world by 2025. In addition, it is reported that the world’s energy consumption will increase by 49% from 2007 to 2035^2,4^.

About 97% of the water that exists on earth is saltwater, while the percentage of freshwater does not exceed 3%. Thus, desalination is considered one of the most common processes to produce freshwater^5,6^. The process of desalination involves taking out salts, other minerals, and impurities from brackish water, saltwater, and wastewater effluent^7^. However, desalination faces some challenges, like high costs, energy intensity, and environmental impacts, and scientists have been doing their best to overcome them in recent years^8^. There are two desalination techniques, namely, thermal and membrane techniques^9^. Thermal desalination, which involves multistage flash, multiple effect distillation, and vapor compression, separates salt from water by evaporation and condensation. Meanwhile, regarding membrane desalination that involves reverse osmosis (RO) and electrodialysis (ED), water diffuses through the membrane, but salts are nearly entirely preserved. Because fossil fuel supplies are freely accessible and the local feed water is of low quality, thermal desalination has remained the predominant method of choice in the Middle East. However, membrane-based desalination processes offer a more viable option to provide fresh water for global water scarcity than thermal-based desalination processes in terms of energy consumption^7^.

Among all membrane-based desalination technologies, reverse osmosis (RO) is the most widespread pressure-driven approach; however, its pressure requirements and hence energy consumption are the highest^10^. Thus, there is a growing demand to develop more energy-efficient desalination processes^11^. Recently, forward osmosis (FO), a relatively novel membrane method, has been widely considered for water treatment and in desalination units^12–14^. FO employs the natural osmotic pressure difference between draw solution (DS) and feed solution (FS) to move clean water across a semipermeable membrane^15,16^. The concentrated fluid that powers the FO process on the permeate side of the membrane is called the osmotic engine, or DS. The feed solution's osmotic pressure should be lower than the DS’s^13,17^. The fundamental advantage of this technology is that it does not necessitate a large amount of energy or electricity, as do other membrane-based techniques. Because it operates without hydraulic pressure, the fouling proclivity is much decreased, allowing for high water permeability and salt rejection^11,18^. However, finding a suitable draw solute (DS) that can produce a high osmotic pressure and is easy to recover or regenerate is still challenging^2^.

There are many types of draw agents used in the FO process: organic draw agents (e.g., oligomers, hydrogels, and different organic solutions) or inorganic draw solutions (e.g., NaCl and ammonium carbonate solutions)^13,19^. Currently, hydrogels have attracted attention as draw agents due to their low toxicity, low reverse solute flux, and high capacity to absorb water^13^. Hydrogels are three-dimensional network structures that are synthesized from synthetic or natural polymers and can absorb a significant amount of water^20^. Stimuli-responsive hydrogels specifically exhibit reverse volume change or solution-gel phase transition owing to external environmental stimuli such as temperature, pressure, pH, light, and solution composition^21,22^. Table 1 shows different examples of hydrogels that have been used as draw agents in FO desalination processes lately and their water flux. To our best knowledge, no previous studies have synthesized a bioartificial hydrogel for the FO process. Bioartificial hydrogels represent a new class of polymeric constituents based on blends of synthetic and natural polymers, designed with the purpose of producing new materials with enhanced properties with respect to the individual components^23^.

**Table 1:** Representation of different types of hydrogels used in FO process.

Hydrogel	Description	Feed solution	Water flux (LMH)	References
P(AMPS-*co*-AM)	Electro-responsive hydrogel composed of acrylamide and 2-acr-ylamido-2-methyl-1-propane sulfonic acid	2000 ppm NaCl	2.76 (initial 1 h)	^20^
(PNIPAM/γ-PGA/PEG)	Thermo-responsive hydrogel prepared from *N*-isopropyl-acrylamide in the presence of polyglutamic acid and pore forming polyethylene glycol	DI water	1.99 (initial 0.5 h)	^28^
		0.05 wt% NaCl	1.65 (initial 0.5 h)	
		0.1 wt% NaCl	1.31 (initial 0.5 h)	
		0.2 wt% NaCl	1.08 (initial 0.5 h)	
SSA-H	Thermo-responsive hydrogel based on sewage sludge ash	DI water	Average water flux of 2.33 in 24 h	^29^
(g-PDMAAm)	Dual CO_2_ and thermo-responsive poly(*N*,*N*-dimethylallylamine) hydrogel	1.75% NaCl	44 (initial)	^30^
P(NIPAM-*co-*DEM)	Thermo-responsive hydrogel prepared by the blend of deep eutectic mixture and *N*-isopropylacrylamide	DI water	2.38 (initial)	^31^
		2000 ppm NaCl	1.81 (initial)	
SA/FG/PEG	Green hydrogel was prepared from a polymer blend of flaxseed gum (FG) and sodium alginate using epichlorohydrin (ECH) as a crosslinker and polyethylene glycol (PEG) as a semi-interpenetrating network polymer	Distilled water	1.27	^32^

The main goal of the present work is the synthesis of a bioartificial hydrogel from a blend of sodium alginate (SA) and polyvinyl alcohol (PVA) using epichlorohydrin (ECH) as a crosslinking agent. Sodium alginate is a natural anionic copolymer composed of two monomer units (1–4) linked β-d-mannuronic acid (M) and α-l-guluronic acid (G) and is extracted from brown algae^24^. Polyvinyl alcohol (PVA) is a long-chain, water-soluble polymer created by hydrolyzing polyvinyl ester (often polyvinyl acetate). It combines the features of rubbers and plastics while also exhibiting special traits such as low cost, low toxicity, great mechanical strength, outstanding biocompatibility, and chemical stability^25^. The aim of this polymer blend is to combine SA's high hydrophilic qualities^26^ to overcome PVA's low responsiveness^25^ and PVA’s strong mechanical capabilities^25^, which are highly recommended properties during swelling and deswelling performances. This is due to the formation of a novel SA/PVA hydrogel with excellent water absorbance and mechanical properties. Epichlorohydrin was selected as a crosslinker because, in a basic medium, it behaves as a bifunctional molecule toward hydroxyl groups^27^, making it an appropriate crosslinking agent between SA and PVA that are rich in OH groups^26,27^.

The effects of the percentage of PVA in the blend and the cross linker/total polymer ratio on equilibrium swelling ratio (ESR) were systematically investigated to optimize the composition of the hydrogel. The water flux and the reversed solute flux of the optimum SA/PVA hydrogel were evaluated in a batch FO unit under the effect of different parameters such as hydrogel particle size, temperature of FS, FS concentration, and membrane orientation.

## Materials and methods

### Materials

Sodium alginate (SA, 96.5%) purchased from OXFORD LAB FINE CHEM LLP (India), and polyvinyl alcohol (PVA, 98–99%, 1700–1800 degree of polymerization) was obtained from LOBA CHEME (India). Sodium hydroxide was provided by Alahram company (Egypt). Epichlorohydrin (ECH, 92.54%) was acquired from LOBA Cheme (India). Acetone (99%) was supplied from ADWIC (Egypt). All chemicals were used as received without any further purification. Cellulose triacetate (CTA) membrane was provided by the national research center (Cairo, Egypt). The specifications of CTA membrane are listed in Table 2. Real brackish water from two different wells (1160.8 and 1633.8 ppm) was obtained from Alamein City, Egypt.

**Table 2:** Specifications of CTA membrane.

Material	Cellulose triacetate membrane
Hydrophilicity	Hydrophilic material
Color	White
Average pore diameter	1.21 nm
Porosity	36%
Tensile strength	33.5 MPa
Elongation	43.8%

### Synthesis of the hydrogel

**Figure 1:**
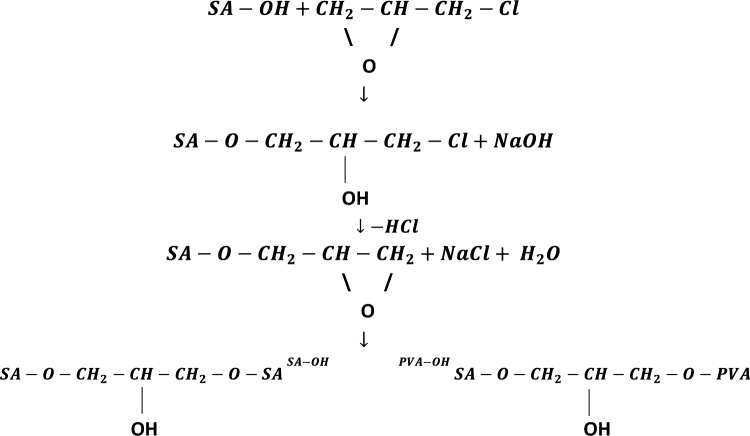
Scheme of possible crosslinking reactions between sodium alginate and polyvinyl alcohol using epichlorohydrin.

A certain amount of SA was dissolved in an 8% NaOH solution, while a certain amount of PVA was dissolved in distilled water by heating at 90 °C under stirring for 30 min. Both polymer solutions were mixed by a mechanical stirrer for 1 h to form a homogeneous solution with a total polymer concentration of 8%. After that, ECH crosslinker was added dropwise to the mixture with continuous mechanical stirring until a homogeneous paste was formed. The hydrogel paste was cured at 75 °C for 9 h. The paste was washed with distilled water several times at 60 °C until reaching a pH value close to 7 to ensure the removal of all unreacted chemicals. Finally, the paste was immersed in acetone to eliminate any traces of ECH. The washed hydrogel was dried at 50 °C until completely dried. Figure 1 shows the scheme of the possible crosslinking reactions. The dry hydrogel was grinded and sieved into different average sizes ranging from 60 to 362.5 m, and then used for characterization and as a draw agent in the FO batch unit. The effect of PVA/total polymer mass percentage (0, 25, 50, 75, and 100) and the effect of cross linker/polymer blend mass ratio (0.8, 1., and 1.4) were investigated.

### Hydrogel characterization

The swelling ratio (SR) is one of the most important parameters for evaluating hydrogels. 0.1 g of each hydrogel with different polymer ratios and crosslinking ratios was immersed in distilled water. The weight of the swollen hydrogel was recorded after 1 and 24 h to obtain the optimum composition of the hydrogel, which would be further characterized. The swelling ratio (SR) of a hydrogel is defined according to the following equation:

1 $$ SR = \left( {W_{s} - \, W_{d} } \right){/}W_{d} $$

where W_s_ is the weight of swollen hydrogel at a room temperature and W_d_ is the weight of the dry hydrogel.

The surface morphology of the optimal hydrogel before and after the swelling was determined using Jeol (JSM-IT200, Japan) electron scanning microscopy. The swollen SA/PVA hydrogel sample for 1 h were quickly frozen in liquid nitrogen and then freeze-dried under vacuum at − 42 °C for 3 days until all the water sublimed. The freeze-dried hydrogel was fractured carefully, and coated with gold for scanning.

The compressive strength of the optimal swollen hydrogel was measured at different distances using MultiTest-5xt (USA). The sample was prepared in a cylinder shape. Then it was immersed in distilled water for an hour. The surface area of the swollen cylinder was 15.89 cm^2^, and its length was 2 cm. The swollen sample was inserted for the compression test at 25 °C.

Wide angle X-ray diffraction profiles (XRD) of PVA, SA and the optimized SA/PVA dry hydrogel powders were determined at room temperature with a X-ray powder diffractometer -XRD-D2 phaser (BRUKER, GERMANY). The 2θ range for the samples was 10°–100°.

Fourier transform infrared spectroscopy (FTIR) (Bruker Tensor 37, Germany) was used to confirm the chemical composition of liquid ECH and SA, PVA, and the optimum SA/PVA solid powder.

### FO batch process

**Figure 2:**
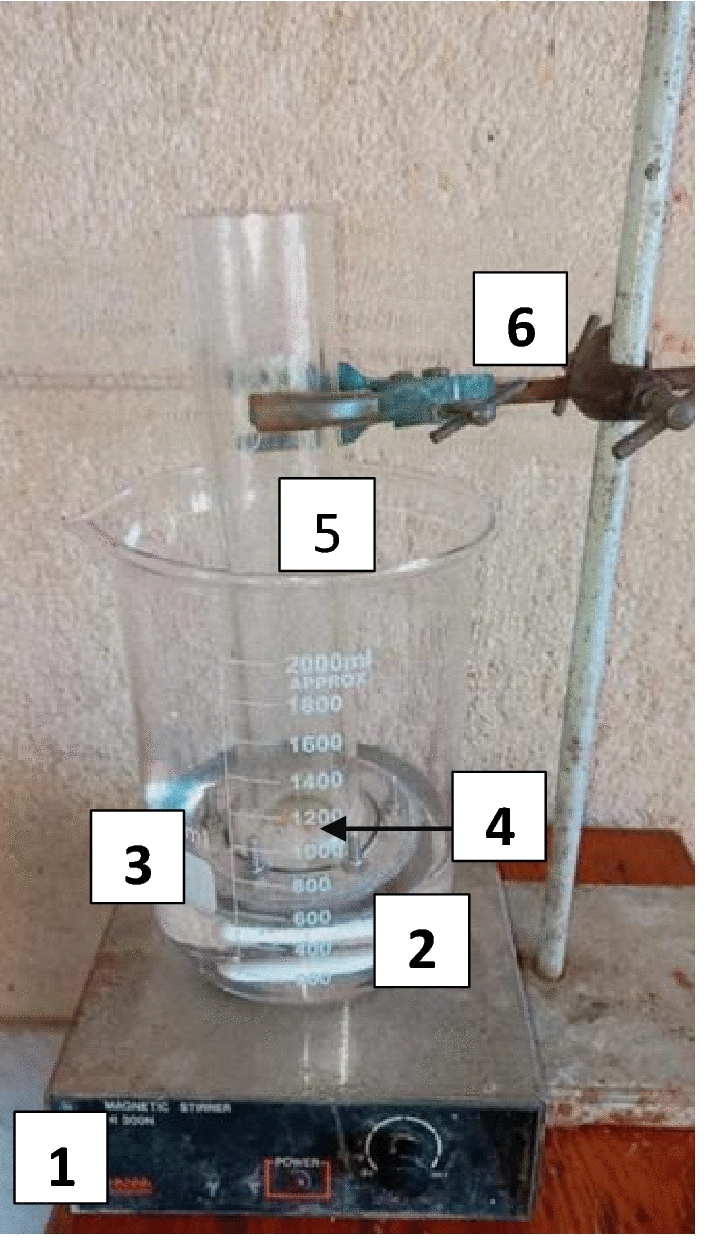
Batch FO set up, where 1—Magnetic stirring device, 2—Magnetic stirring rod, 3—Beaker, 4—Hydrogel covers the top side of the membrane, 5—FO cell, and 6—Holder.

Figure 2 shows the main components of the FO batch setup used in the present study. Before each test, the FO cellulose triacetate membrane was soaked in the feed solution, and the system was conditioned for about 1 h. At the beginning, the top side of the membrane was covered by a very thin layer of ground dry hydrogel of a certain average particle size. The membrane was immersed in a beaker containing the feed solution. The conductivity of the FS was measured by (DiST4, HI98304, Romani), and the weight of the hydrogel was recorded before and after the run. Temperature, FS concentration, average particle size of the hydrogel, and membrane orientation were the main parameters studied in this FO process. In all FO experiments, the time of the process was an hour, the membrane effective area was 12.56 cm^2^, and the powder area density was 0.016 g/cm^2^. The performance of the hydrogel as a draw solute was expressed in terms of water flux, which was calculated according to the following equation:

2 $$ J_{w} = (W_{s} {-} \, W_{d} ){ /}\rho_{w} At $$

where ${{\text{J}}}_{{\text{w}}}$ is the water flux through FO membrane; ${{\text{W}}}_{{\text{s}}}$ and ${{\text{W}}}_{{\text{d}}}$ are the weight of the swollen hydrogel and the dried sample respectively; ${\uprho }_{{\text{w}}}$ is the density of the water; A is the surface area of the membrane and t is the time of the process^33^.

## Results and discussion

### Hydrogel characterizations

#### Factors affect swelling ratios of the hydrogel

The swelling of the polymer hydrogel is driven by the osmotic pressure originating from the dissociation of the ionic groups and the solvation force generated by the hydrogen bonding interaction between the hydrogel network and H_2_O. It is strongly affected by the polymer content and the degree of crosslinking^34^.

**Figure 3:**
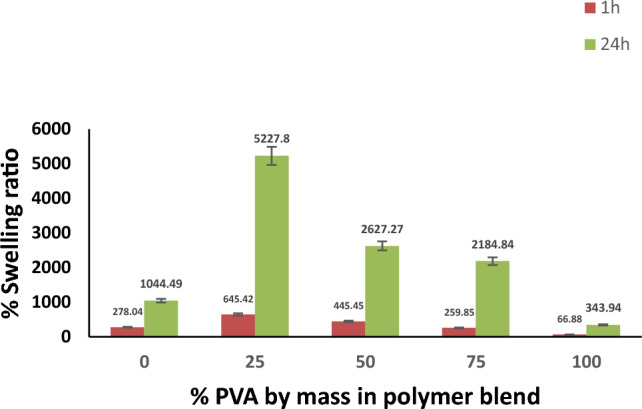
Effect of %PVA by mass in polymer blend on %swelling ratio at different time intervals.

Figure 3 exhibits the effect of different PVA mass percentages in the polymer blend on the swelling ratio (%) measured at two different time intervals. It is clear that raising the PVA content in the polymer blend has a negative effect on the swelling measurements of the hydrogel. Thus, a hydrogel made of 100% PVA has the lowest swelling ratio. These results are largely consistent with previous studies^35,36^. This low swelling response of PVA can be explained by its high degree of crystallinity^36^. PVA hydrogels typically have a porous structure, with pores filled by a polymer-poor phase. This phase is structured and comprises of tiny micellar crystalline aggregates of PVA chains and amorphous domains. The solvent swells the PVA chains in the amorphous domains, which act as tie chains that connect the fringed micelle-like crystals. Thus, the high degree of crystallinity has a negative effect on the hydrogel’s swelling measurements^37^.

It is also obvious from Fig. 3 that by reducing the PVA content in the polymer blend from 100 to 25%, or, in other words, by increasing the SA (%) in the polymer blend from 0 to 75%, the swelling ratio upgraded from 66.88 to 645.42 after 1 h and from 343.94 to 5227.8 after 24 h. This is due to the presence of carboxyl groups in the backbone of SA, which generates high osmotic pressure due to their dissociation, which contributes to the high swelling of the hydrogel^26,38^. From the studied PVA percentages, the optimal PVA (%) in the polymer blend was 25%.

**Figure 4:**
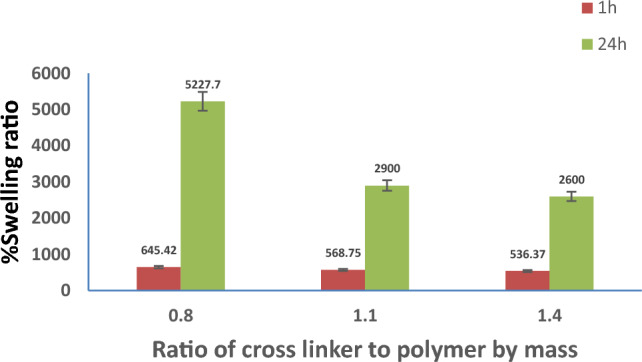
Impact of cross linker/polymer blend mass ratio on %swelling ratio at different time intervals.

Figure 4 shows the effect of the crosslinker/polymer blend mass ratio on the swelling ratio (%) at two different time intervals. It is well noted that as the dosage of the crosslinker increases, the swelling ratio (%) decreases. This is because the polymer chains would be further crosslinked, and the polymer networks would become more compact. Consequently, it is difficult for water molecules to penetrate into the polymer networks, resulting in low SR^39^. In addition, according to the crosslinking reaction that is presented in Fig. 1, some of the hydroxyl groups in SA and PVA are consumed in the crosslinking reaction. These groups, besides carboxyl groups, are the hydrophilic groups, which are responsible for hydrogen bond formation with water molecules. Thus, the high crosslinking density means more consumption of OH groups, and consequently, the swelling measurements will be reduced^40^. Accordingly, the optimal ECH/total polymer blend mass ratio was 0.8.

From all these swelling measurements, the optimum hydrogel’s composition is 25% PVA by mass in the polymer blend and 0.8 ECH/total polymer blend by mass. This optimal composition would be considered for all further characterizations and as a draw agent in the FO cell.

#### SEM

**Figure 5:**
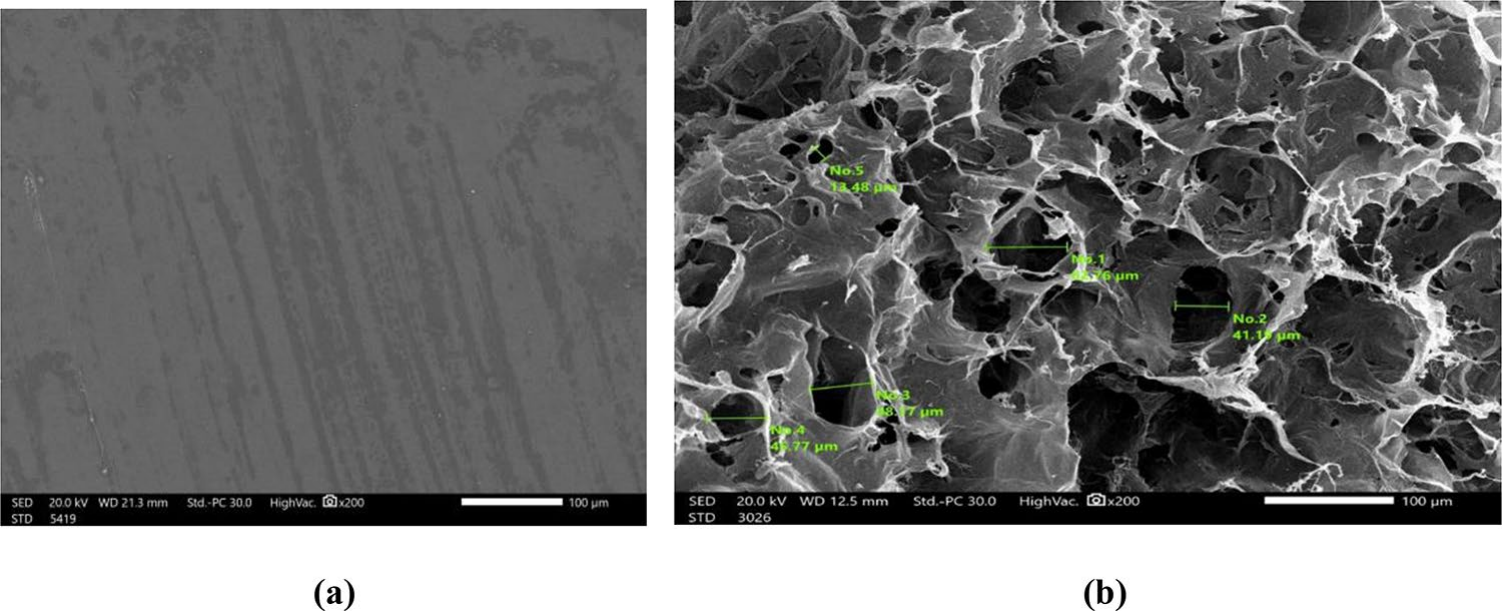
Surface morphology (× 200 magnification) of optimum (**a**) dry hydrogel and (**b**) swollen hydrogel.

The porosity of hydrogels influences their water absorption. As a result, hydrogel microstructure morphology is a significant property^41^. Figure 5 shows two images of the prepared optimum hydrogel before and after swelling to observe the surface changes as a result of swelling. After swelling, the hydrogel is freeze-dried for observation of pores that were previously filled with water molecules^42^. Figure 5b represents the well-defined, interconnected, three-dimensional porous network structure of the swollen hydrogel. Figure 5b shows a great expansion in the pore structure when compared with the image of the hydrogel in the dry state in Fig. 5a. This may be attributed to the fact that SA acted as an expander of the pore size because of its high absorbency for water^26^. Actually, these results can be ensured by the swelling measurement of the pure SA hydrogel, which was four times higher than that of the pure PVA hydrogel after 1 h, and it increases by upgrading the SA content. In addition, as mentioned in the previous literature, the pore size of pure PVA hydrogel ranges from 2 to 7 μm^43^, while the average pore size of the present hydrogel is 42 μm. Thus, the incorporation of SA in the hydrogel successfully enhanced the pore structure of the hydrogel.

#### Compressive strength test

**Figure 6:**
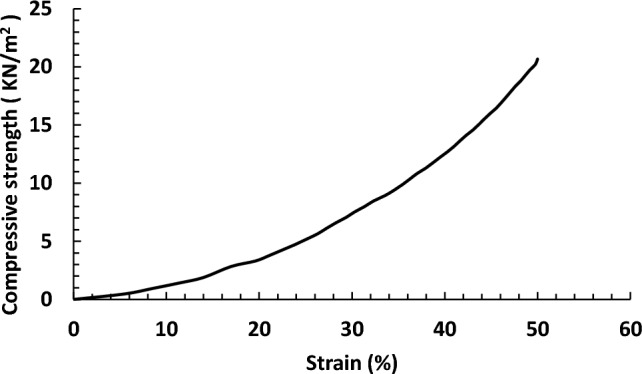
Representation of the compression strength test results of the optimum swollen hydrogel.

Figure 6 shows the results of the compression strength test of the swollen SA/PVA hydrogel. It is clear that the swollen SA/PVA hydrogel can be compressed to its half-length at a compressive strength of 20.67 KN/m^2^ without any deterioration in its shape. This test is beneficial for the water recovery process, which could be detailed in a separate published work in the future. This excellent mechanical property is due to two main reasons: Firstly, the incorporation of PVA in the polymer blend, which gathers the characteristics of rubbers and plastics, has a positive effect on the mechanical stability of the hydrogel^25^. Secondly, the chemical crosslinking using ECH as the chemical crosslinker enhances the mechanical performance of the hydrogel, especially when it is compared to physically crosslinked hydrogels^44^. Al-Sabagh et al. proved that fact by preparing PVA hydrogels using different crosslinking mechanisms. They found that the ECH crosslinker exhibited the best mechanical performance of the hydrogel in the wet state when compared to the physically crosslinked hydrogel or even the chemically crosslinked hydrogel using glutaraldehyde^45^. Elasticity is the ability of a material to attain its original shape and dimension after the removal of a load. Thus, it is a very essential mechanical property to be studied, especially since a hydrogel composed of a polymer network uses the concept of originally developed rubber elasticity^46^. The calculated modulus of elasticity of our optimum hydrogel is 12.19 kPa. The elasticity of a hydrogel is dependent on its crosslinking density. This is because higher crosslinking ratios than optimal make the contact area between polymer chains greater, and the structure will be denser and more compact. This results in less elastic and more brittle material^47^. So, we can consider this calculated modulus to be an acceptable value, as it is the value of the optimum crosslinked hydrogel.

#### FTIR

**Figure 7:**
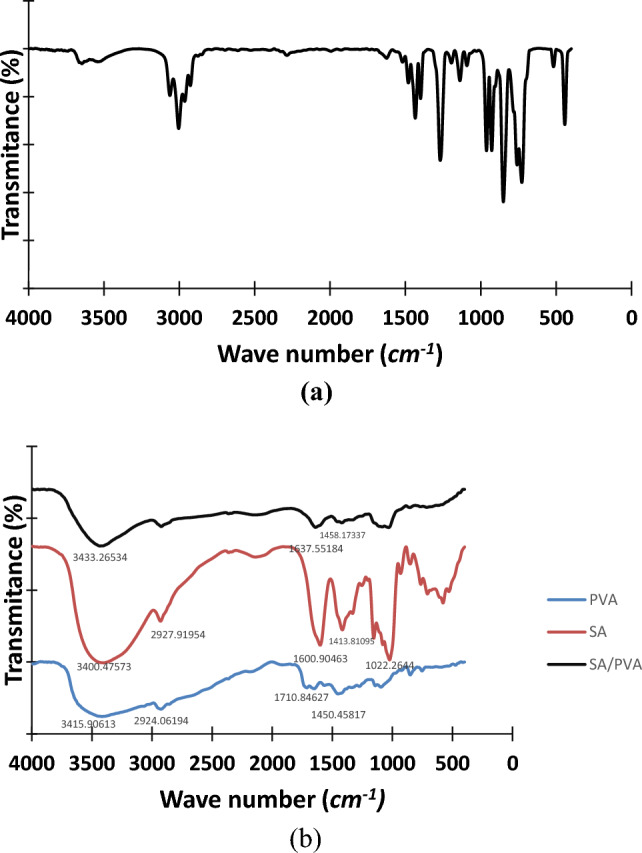
FTIR spectrum of (**a**) ECH and (**b**) SA, PVA and optimum SA/PVA hydrogel.

Figure 7a exhibits the IR spectrum of epichlorohydrin. The vibrations at 962 and 925 cm^−1^ are characteristics of C–C symmetric deformation of the epoxide functions of epichlorohydrin^48^.

Figure 7b shows the IR spectrum of sodium alginate, polyvinyl alcohol, and the optimum SA/PVA dry hydrogel. For SA, the broad peak at 3400.47 cm^−1^ corresponds to intra and intermolecular hydrogen bonds from OH stretching. Peaks around 2927.91 cm^−1^ may be due to the C–H stretching vibrations. The two peaks at 1600.90 cm^−1^ and 1413.8 are due to C=O asymmetrical and symmetrical stretching vibrations of carboxylate salt group. Whereas the peak at 1022.26 cm^−1^ is attributed to the stretching vibration of C–O–C groups^24,49^. For PVA, the broad and strong peak appears at 3415.9 cm^−1^ is a characteristic peak for the stretching –O–H group. The peak at around 2924.06 cm^−1^ belongs to saturated C–H stretching, whereas the peak at 1450.458 cm^−1^ is related to –CH2– bending from the alkyl group. The spectrum exhibits a band in the region of 1710.84 cm^−1^, which corresponds to the residual acetal group stretching. This is related to the partial (87–89%) hydrolyzation of the PVA. Moreover, the peak at 1095.41 cm^−1^ is due to the C–OH stretching of PVA^49,50^. It is well obvious that the peaks corresponding to –OH stretching are shifted to 3433.26 cm^−1^. In addition, the two peaks belonging to the C=O asymmetrical and symmetrical stretching vibrations of the carboxylate salt group are shifted to 1637.55 and 1420.75 cm^−1^ respectively. The peak that characterizes acetal group stretching disappeared, which may be due to the hydrolysis side reaction during crosslinking. The peak corresponding to –CH_2_– bending from the alkyl group is shifted to 1458.17 cm^−1^. The vibrations at 962 and 925 cm^−1^, which are characteristics of the epoxide functions of epichlorohydrin, have disappeared. The disappearance of such peaks indicates the epoxide ring opening due to the crosslinking reaction. All these observations confirm the chemical reaction between PVA and SA using ECH.

#### XRD

**Figure 8:**
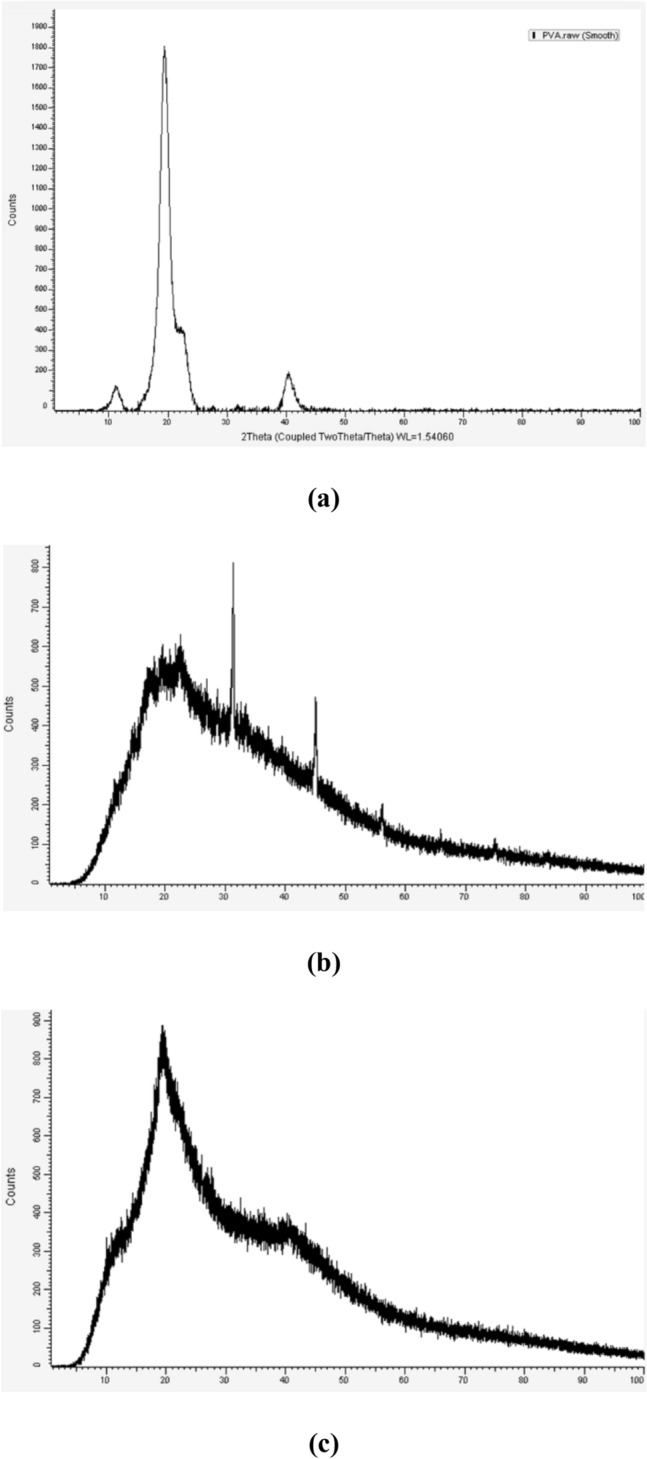
XRD spectrum of (**a**) PVA, (**b**) SA and (**c**) optimum SA/PVA hydrogel.

The XRD spectra of PVA, SA, and the optimum SA/PVA dry hydrogel are shown in Fig. 8. It can be seen that the diffraction patterns of PVA have a strong crystalline reflection at 2θ = 20° and 23°, which are characteristic of PVA and represent reflections from (1 0 1) and (1 0 1′) from a monoclinic unit cell^51^. Whereas, the XRD of the SA sample presented two weak peaks and broad diffractions at 15.0° and 22.1°, indicating a rather amorphous structure^52^. The XRD pattern of the SA/PVA hydrogel blend shows no sharp peaks and a broad diffraction at 2θ = 20°, which indicates an amorphous structure. The disappearance of sharp peaks and weak peaks related to PVA and SA, respectively, confirms the presence of crosslinks between the PVA and SA chains, which result in the amorphous structure of the SA/PVA hydrogel blend.

### FO performance evaluation

#### Membrane orientation

In the preliminary experiments using distilled water as feed solution, when FO mode (support layer facing draw agent) was applied in the FO batch setup, it was found that the support layer was clogged with the hydrogel’s particles. This will lead to many consequences, including a reduction in the membrane’s lifetime and a loss of the hydrogel’s mass. In addition, it is considered a resistance to the water flow on the permeate side, which consequently reduces the achieved water flux. So, all the following FO runs were carried out by applying PRO mode (active layer facing draw agent). In addition, according to most of the previous works, adjusting membrane orientation in PRO mode is preferred rather than FO mode during the FO desalination of synthetic saline water and brackish water to avoid internal concentration polarization, which can diminish water flux by more than 80% of its value^53–55^. Thus, the effective osmatic pressure driving force in the PRO mode is higher than the effective osmatic pressure driving force in the FO mode^56^.

#### Average hydrogel particle size

**Figure 9:**
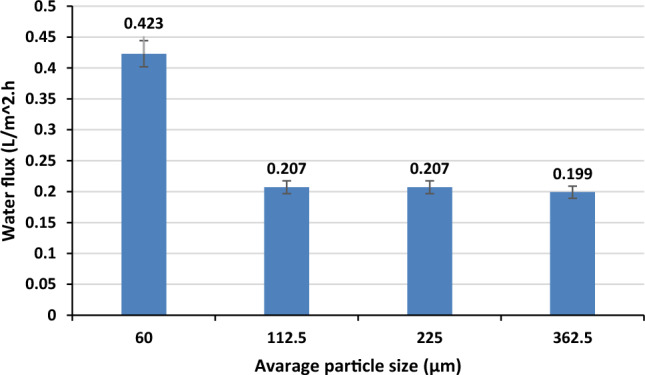
Effect of average hydrogel particle size on water flux (distilled water as FS, and FS temperature at 25 °C).

The average hydrogel particle size has a significant role in the performance of the FO process. Figure 9 shows the average water flux at different average hydrogel particle sizes. The water flux was reduced to a quarter of its value by increasing the average hydrogel particle size from 60 to 362.5 µm. Thus, it should be pointed out that larger hydrogel particle sizes have a negative effect on water flux. This may be attributed to two main facts: (i) The smaller size of the hydrogel particle can lead to a higher contact area between the FO membrane and the hydrogel. Thus, the hydrogel will be able to absorb a larger amount of water^57^. (ii) According to gel dynamics theory, the hydrogel swelling rate is inversely proportional to the square of the hydrogel size and proportional to the hydrogel diffusion coefficient. So, water flux will be increased at smaller hydrogel particle sizes^56^.

#### FS temperature

**Figure 10:**
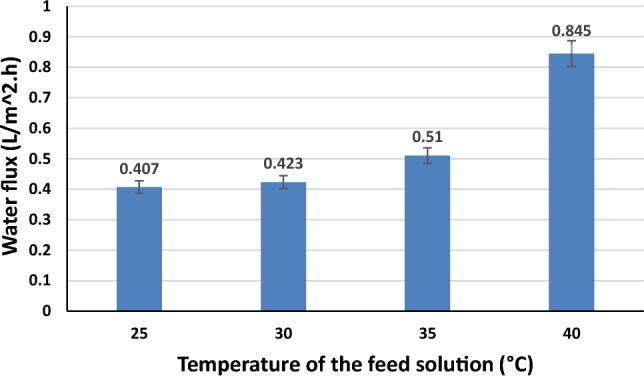
Effect of feed solution temperature on the water flux (distilled water as FS, and optimum hydrogel of 60 µm average hydrogel particle size).

Figure 10 shows the effect of FS temperature on water flux. The water flux was nearly doubled by increasing the FS temperature from 25 to 40 °C. So, it is clear that temperature has a positive effect on water flux. This is because when the temperature of the feed solution increases, its viscosity decreases with a consequent increase in the diffusion coefficient, according to the Stokes–Einstein equation^58,59^. Subsequently, the diffusion of FS from the FS side to the DS side will be enhanced. These results are in agreement with an earlier study^60^. In addition, as it is studied in previous articles, the increase of the FS temperature within range of 20–40 °C has a negligible impact on the CTA membrane structural parameter (S). So, the membrane polymer structure will be conserved without any deterioration during processing at FS temperature up to 40 °C. Moreover, the water permeability (A) parameter will be enhanced by increasing the FS temperature up to 40 °C^61^. We can conclude from our practical results and previous published work that the optimum FS temperature is 40 °C.

#### FS concentration

**Figure 11:**
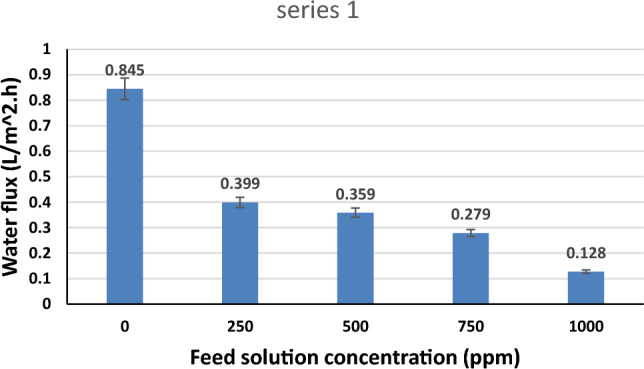
Effect of feed solution concentration on water flux (FS temperature of 40 °C and optimum hydrogel of 60 µm average hydrogel particle size).

Different concentrations of sodium chloride solutions were examined as feed solutions in the FO unit. Figure 11 exhibits the effect of FS concentration on the water flux. As the FS concentration increases from 0 to 1000 ppm, the water flux declines from 0.845 to 0.12 LMH. The present trend can be attributed to the fact that, as the ionic strength of FS increases, the osmotic pressure driving force between the hydrogel and FS decreases, which results in reduced water flux. The present results are consistent with previous studies^28,62^.

Additionally, real brackish water obtained from two different wells with total dissolved solids of 1160.8 and 1633.16 ppm was desalinated by this FO batch system and revealed a water flux of 0.21 and 0.34 LMH, respectively, under optimum operating conditions. From Fig. 11, the achieved water flux at the same operating conditions was 0.128 LMH when a synthetic NaCl solution of 1000 ppm concentration was applied. It is well remarkable that the achieved water flux by the real brackish water is slightly higher, and it increases by raising its TDS. This can be explained by the reduced viscosity of the real brackish water by heightening its TDS, which leads to lower resistance to water flow, and water flux will hence increase. In general, any real brackish water is composed of the dissolution of a variety of salts, such as sodium chloride, potassium chloride, and so on. However, some salts (for example, potassium chloride) reduce the viscosity of water. Actually, the effect of such salt on the viscosity is limited but positive, and its impact is strongly notable at the low TDS of the brackish water^63^.

Table 3 shows a brief presentation of the FO performance of our work and a previous study that applied a novel hydrogel as a draw agent for FO desalination. This earlier work depended on the incorporation of PVA as a semi-interpenetrating network polymer (semi-IPN) with N-isopropylacrylamide (NIPAm) to form a semi-IPN thermally responsive hydrogel.

**Table 3:** FO performance of the present work and previous studies.

Hydrogel	Average particle size (µm)	Membrane	Temperature (°C)	FS concentration (ppm)	Water flux (LMH)	Reference
PNIPAm-INP-PVA	100	CTA	25	2000 (NaCl)	0.12 (1 h)	^64^
SA/PVA (present work)	60	CTA	40	Distilled water	0.845 (1 h)	
				250	0.399 (1 h)	
				500	0.359 (1 h)	
				750	0.279 (1 h)	
				1000	0.129 (1 h)	

#### Reverse solute flux

Reverse solute flux (RSF) is defined as the solute diffusion from the draw solution side to the feed solution side because of the concentration gradient across the membrane, which consequently has a negative effect on the driving force in the FO process^65^. In the present work, a hydrogel is used as a draw agent that is a solid material with a high water absorption capacity, so there is no concentration gradient, and hence reverse solute flux is negligible in all FO experiments^66^. The insignificant RSF was confirmed by conductivity measurements of distilled water, which was applied as FS in FO experiments. Typically, conductivity measurements of distilled water provide the most accurate indication of RSF as it achieves the highest driving force when compared to other feed solutions with higher concentrations. The conductivity of the distilled water before and after the FO run was recorded at 0.01 µS/cm, which means that there was no RSF accomplished. These results are comparable to those of previous studies mentioned in Table 1.

### The contribution of the present work

This framework introduces a novel hydrogel that was synthesized from a polymer blend of SA and PVA, and ECH was used as a crosslinker. Researchers can utilize this cutting-edge hydrogel in various applications due to its superior swelling measurements and mechanical properties. In this work, we examined its performance as a draw agent in forward osmosis desalination at a wide range of average hydrogel particle size, FS temperature, and concentration. In addition, its behavior against real brackish water was investigated. The results were reasonable, as they were described above. According to our results, this hydrogel is suitable for the desalination of multiple categories of mild saline water. However, we are working now to improve its performance to make it appropriate to desalinate harder salt water.

## Conclusions

An innovative bioartificial hydrogel was successfully synthesized from a blend of SA and PVA using ECH as a crosslinker. This hydrogel was characterized by swelling ratio, FTIR, SEM, and XRD. The performance of this hydrogel as a draw agent was examined in the FO process through different parameters, including hydrogel particle size, temperature and concentration of the feed solution, and membrane orientation. Brackish water with different concentrations of 1160.8 and 1633.16 ppm was also examined as a feed solution. The conclusions of the present work can be summarized as follows:

- The optimum hydrogel with a crosslinking ratio of 0.8 and 25% PVA achieved an equilibrium swelling ratio (%) of 5228.
- When distilled water was utilized as the FS, average hydrogel particle size was 60 µm, and the FS temperature was 40 °C, the maximum water flux of 0.845 LMH was attained.
- Real brackish water from two different wells with total dissolved solids of 1160.8 ppm and 1633.16 ppm revealed a water flux of 0.21 and 034 LMH, respectively.
- Reverse solute flux was negligible in all FO experiments.

## Data Availability

All data and materials used in current research are available in this manuscript.
